# Gas-phase, conformer-specific infrared spectra of 3-chlorophenol and 3-fluorophenol†

**DOI:** 10.1039/d4cp04352a

**Published:** 2025-02-20

**Authors:** Olga A. Duda, Gerrit C. Groenenboom, Daniel A. Horke, Joost M. Bakker

**Affiliations:** a Institute for Molecules and Materials, HFML-FELIX Laboratory, Radboud University Toernooiveld 7 6525 ED Nijmegen The Netherlands joost.bakker@ru.nl; b Institute for Molecules and Materials, Radboud University Heijendaalseweg 135 6525 AJ Nijmegen The Netherlands

## Abstract

Conformational isomerism of phenol derivatives has been a subject of extensive spectroscopic study. Combining the capabilities of the widely tuneable infrared free-electron laser FELIX with molecular beam technologies allows for revisiting existing data and gaining additional insights into far-IR spectroscopy of halogenated phenols. Here we present conformer-resolved infrared spectra of the *syn* and *anti* conformers of 3-chlorophenol and 3-fluorophenol recorded *via* IR-UV ion-dip spectroscopy. The experimental work is complemented by density functional theory calculations to aid in assignment of the observed bands. The experimental spectra for the two conformers of each molecule show overall a great similarity, but also include some distinct conformer-specific bands in the spectral range investigated. Our spectra confirm previously reported OH torsional mode frequencies for the *syn* and *anti* conformers of 3-chlorophenol (3CP) at 315 cm^−1^, (Manocha *et al.*, *J. Phys. Chem.*, 1973, **77**, 2094) but reverse their assignment of the 311 and 319 cm^−1^ bands for 3-fluorophenol. 1D torsional mode calculations were performed for 3CP to help assign possible OH torsion overtones.

## Abstract 2

Conformer-specific IR spectra of the *anti* and *syn* conformers of halogenated phenol derivatives clearly show several bands with isomer-specific frequencies, despite the only structural difference between the conformers being the OH group orientation.

## Graphical abstract

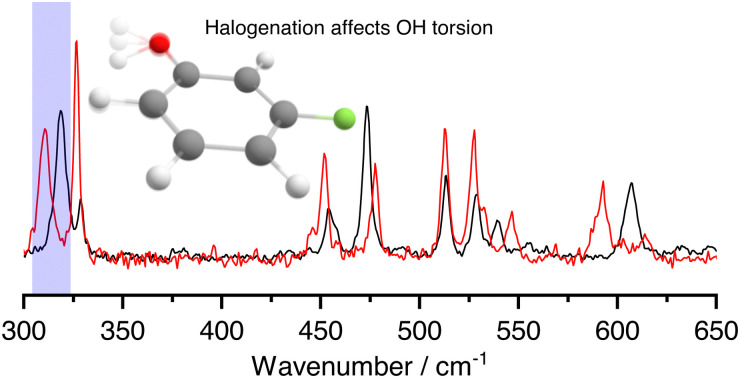

## 1 Introduction

Conformational isomerism plays a key role in numerous aspects of chemistry. In a variety of reactions the conformation of reagents determines their reactivity and intermediates, as well as influences the identity of the products formed.^1–3^ In biological systems, conformation plays a crucial role in enzyme activity ranging from biosynthesis to protein activity regulation.^4,5^ Furthermore, conformational preferences and switching are subjects of interest in macrocyclic and organometallic chemistry due to their potential synthetic and medicinal applications.^6–8^

**Fig. 1:**
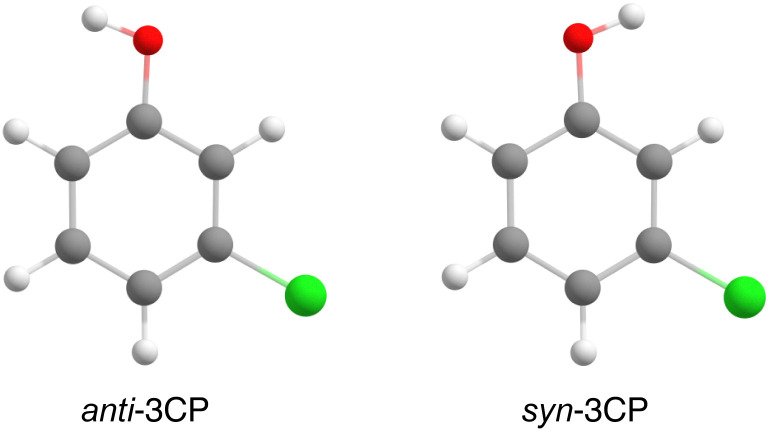
Structures of *anti* and *syn* 3-chlorophenol (3CP) optimised at the B3LYP/def2-TZVP level of theory. The exact *xyz* coordinates for the optimised structures of both 3CP and 3-fluorophenol (3FP) can be found in the ESI.†

To understand this class of reactions on a fundamental level, *ortho*- and *meta*-monosubstituted phenol derivatives are widely studied. Substitution of the hydrogen atom at the *meta* (3) or *ortho* (2) position of phenol leads to *syn*–*anti* conformational isomerism where the hydroxyl group either points towards (*syn*) or away (*anti*) from the heteroatom substituent (Fig. 1). Gas-phase spectroscopic studies allow for a detailed insight into the structural and energetic differences without the additional level of complexity arising from the solvent contribution.^9,10^ Moreover, short-pulse laser systems in conjunction with conformer-specific detection schemes could allow for highly controlled experiments studying the dynamics of interconversion reactions.^11^

The simplest of such systems, halogenated phenols, have attracted considerable spectroscopic attention, with a number of studies focusing on 3-fluorophenol (3FP),^12–18^ and 3-chlorophenol (3CP).^12,13,19–22^ Among these, Manocha *et al.* observed a separation of the OH torsional band in the vapour phase IR absorption spectrum of 3FP, but only a single band for 3CP.^12^ This suggests that the frequency of the torsional mode of 3FP is conformer-specific. For 3CP the frequencies could be near-degenerate, or the population of one of the conformers strongly dominated under the experimental conditions. In the cold environment of a molecular beam, where significantly higher resolutions are achieved, such relative populations can be inferred from the intensities of the origin bands in (1 + 1) UV resonance-enhanced multiphoton ionization (REMPI) spectra. Roughly equal populations of the two 3FP conformers were reported,^13,14^ strengthening the suggestion of a conformer-specific frequency of the torsional mode. Similarly, roughly equal intensities of the *syn* and *anti* origin bands were reported for 3CP,^19,23^ pointing to near-degeneracy of the torsional frequencies.

The OH torsional modes are of particular interest as, in theory, the two conformers of 3-substituted halophenols could be interconverted by a 180° rotation of the hydroxyl group. However, since the frequencies of these modes are found around 300 cm^−1^, they prove challenging to study using traditional tabletop IR lasers.^24^ The far-IR range of the electromagnetic spectrum can readily be accessed with an infrared free-electron laser, such as FELIX.^25^

The low conformational isomerization barrier makes 3FP and 3CP ideal candidate systems for dynamics experiments aimed at directly observing isomerization. Here, interconversion would be triggered by laser excitation of one conformer, and the interconversion processes followed by selectively probing the nascent population of the other conformation. In this light, recent experiments by Lopes Jesus *et al.* are of interest.^17^ They reported the infrared signatures of conformational changes induced in 3FP isolated in argon and nitrogen matrices by non-conformer-specific, broadband IR irradiation. For more controlled experiments aimed at studying interconversion, it is imperative to have detailed information on the conformer-specific vibrational modes. Gas-phase IR-UV ion-dip spectroscopy, first introduced by Page *et al.*,^26^ allows to record conformer-specific infrared spectra by virtue of subtle differences in the conformers' REMPI spectra.^27–29^ In this way, differences in conformer-sensitive vibrational modes can be detected unambiguously. This technique could also confirm the previous assignments of the REMPI spectra to the different conformers.

Here, we present IR-UV ion-dip spectra for the *syn*- and *anti*-conformers of 3CP and 3FP. They are recorded employing the FELIX free-electron laser in the 230–1750 cm^−1^ (3CP) and 270–1750 cm^−1^ (3FP) spectral ranges. The spectra observed are complemented by Density Functional Theory (DFT) calculations to assign the vibrational bands. A particular focus is placed on finding the OH torsional mode as it is the coordinate along which conformational interconversion is likely to take place.

## 2 Methods

3CP and 3FP (99% purity, Sigma Aldrich) were heated to 60–75 °C and seeded into Ar carrier gas at a stagnation pressure of 1–3 bar. The gas mixture was expanded into vacuum (1 × 10^−7^ mbar background pressure, 5 × 10^−6^ mbar when operational) through a pulsed General Valve, with the nozzle held at 10 °C above the sample temperature to prevent condensation. The molecular beam formed was collimated using a 2 mm diameter skimmer (Beam Dynamics, Inc.) upon entering a differentially pumped vacuum chamber. Here, it was illuminated by counter-propagating IR light produced by the free-electron laser FELIX.^25^ The package of molecules illuminated was then allowed to propagate several tens of μs until it reached the extraction region of a reflectron time-of-flight mass spectrometer (TOF-MS) (Jordan TOF Products, Inc.), where it was intersected by the frequency-doubled output of an Nd:YAG-pumped dye laser (Lioptec LiopStar, Coumarin 153 dye, ∼1 mJ per pulse). All ions formed were mass-separated by the reflectron TOF-MS and detected using a microchannel plate. The UV laser was tuned to the 0–0 transitions (S_1_ ← S_0_) of the *syn*- and *anti*-conformers of 3CP (35 783, 35 902 cm^−1^) and 3FP (36 632, 36 839 cm^−1^)^13,14^ producing a constant ion signal. When the IR laser was in resonance with an IR-active vibrational mode of the species of interest, depopulation of the vibrational ground state in the electronic ground state occurred. This, in turn, led to depletion in the ion signal. The experiment was run at twice the FELIX repetition rate of 5 Hz, enabling the recording of reference mass spectra to compensate for long-term source fluctuations. The spectra presented were constructed using the depletion yield, defined as the logarithmic ratio of the ion intensity without and with IR irradiation, and normalized by the macropulse energy. The spectra presented for 3CP are isotope-selected for the more abundant ^35^Cl-substituted isotopomer by virtue of employing mass spectrometry for detection.

The FELIX light is formed in a 10 μs pulse train (macropulse) consisting of 1-ns spaced picosecond pulses (micropulses). Macropulse energies used ranged from ∼70 mJ per pulse in the 1800 cm^−1^ region to ∼20 mJ around 300 cm^−1^. The laser is near-transform-limited and tuned to a spectral bandwidth of 0.4% full-width at half-maximum (FWHM) of the central frequency. The IR wavelength was calibrated using a grating spectrometer.

DFT calculations were performed using the Gaussian 16 suite^30^ with the B3LYP functional^31^ and the def2-TZVP basis set.^32^ For the optimized structures, harmonic frequencies were calculated to ensure true minima, as well as to compare to the experimental spectra. For the latter purpose, the calculated harmonic frequencies are scaled by an empirical factor of 0.985 to compensate for anharmonicities.^33^ The scaled stick spectra were subsequently convolved with a Gaussian lineshape function with a 0.4% FWHM, mimicking the FELIX spectral bandwidth. All harmonic frequency values used in this work are scaled values. Because low-frequency vibrations of phenol derivatives exhibit a significant degree of anharmonicity,^10,24^ additional anharmonic frequency calculations were carried out utilizing the Rayleigh–Schrödinger perturbation theory.^34^ To identify possible overtones of the torsional modes of 3CP, the torsional coordinate was scanned using Gaussian *IRC* keyword. The first order nature of the transition state was ensured by calculating harmonic frequencies and finding a single imaginary frequency. The resulting potential was used to calculate the torsional vibrational wavefunctions and frequencies.

## 3 Results and discussion

**Fig. 2:**
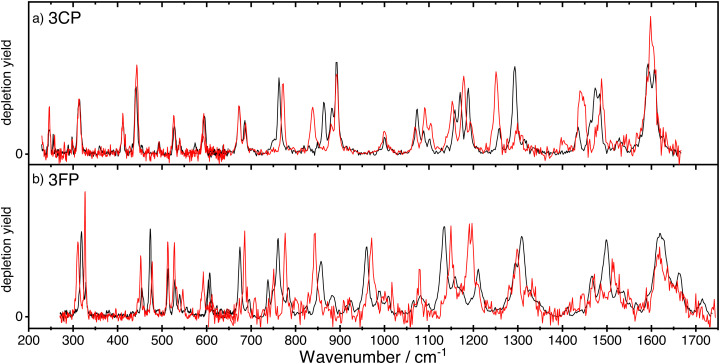
The experimental IR ion-dip spectra of the *anti*-(black curve) and *syn*-(red curve) conformers of 3CP (panel a) and 3FP (panel b). The spectrum of *syn*-3FP was multiplied by a factor 2 in the region above 600 cm^−1^ to facilitate comparison between the two conformers.

The spectra of *anti*- and *syn*-3CP and 3FP are shown together, respectively, in panel (a) and (b) of Fig. 2, allowing for a direct comparison. The depletions observed and the signal-to-noise of the spectrum for *syn*-3FP in the higher frequency range are significantly lower than those for *anti*-3FP. To aid the comparison, the *syn*-3FP spectrum is vertically scaled in the 700–1700 cm^−1^ range. We speculate that this part of the spectrum is recorded with a smaller spatial overlap, because the calculated spectra (discussed in Sections 3.1 and 3.2) suggest no large differences between intensities. The unscaled *syn*-3FP spectrum is shown in Fig. 2 of the ESI.† The 3CP spectra are presented without scaling.

All spectra for 3CP and 3FP exhibit numerous distinct bands, with the spectral bandwidth progressively growing with increasing wavenumber, reflecting the FELIX spectral bandwidth. Although IR ion-dip spectroscopy is widely assumed to be a single-photon technique, we cannot rule out multiple photon absorption, especially for some of the stronger bands that may suffer from saturation and additional (power) broadening. Differences in both position and intensity are observed between the two conformers in the case of 3FP across the entire spectral range. In contrast, below 700 cm^−1^ the spectra of the 3CP conformers are near identical with marked differences becoming significant in the higher frequency range.

### 3.1 Spectroscopy of 3-chlorophenol

**Fig. 3:**
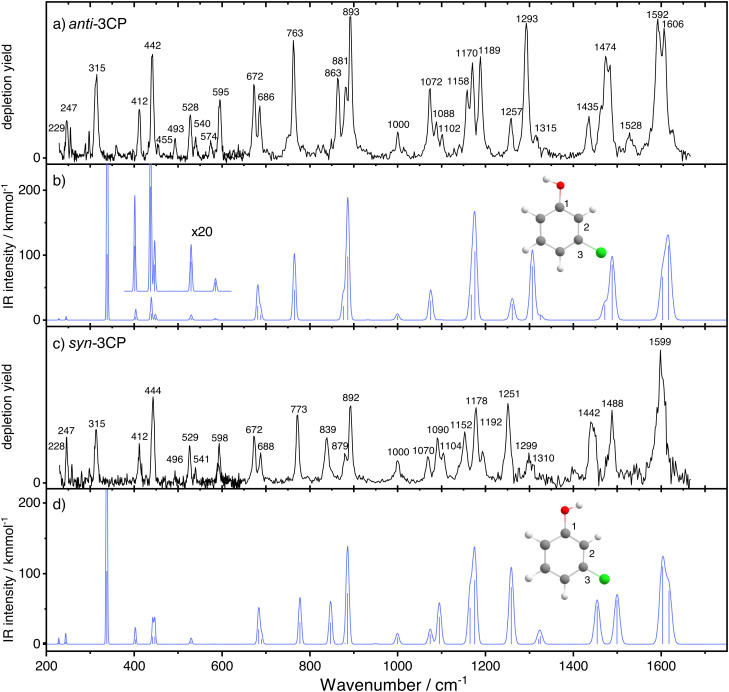
The experimental (a) and (c) and simulated (b) and (d) spectra of *anti*- and *syn*-3CP, respectively. In panels (b) and (d), the blue line represents the scaled harmonic spectrum.

Fig. 3 shows the conformer-specific infrared spectra recorded for *anti*- (panel a) and *syn*-3CP (panel c). Each experimental spectrum is compared to a DFT-calculated spectrum in the harmonic approximation in panels (b) and (d). Overall, the harmonic calculated spectra are in good agreement with the experimental spectrum of *syn*- and *anti*-3CP, even to the extent that most differences between the experimental spectra for each conformer are faithfully reproduced. Below, we globally discuss the assignments of the bands in the experimental spectra, with a special focus on where the two experimental spectra differ. A detailed assignment of all bands is found in Table 1. The in-text discussion uses an *anti*/*syn* convention when stating the mode frequencies.

**Table 1:** Observed (*ν̃*_exp_) and calculated (*ν̃*_DFT_) band frequencies in cm^−1^ in the IR-UV ion dip spectrum of 3-chlorophenol and their assignments. For vibrations which are analogous to phenol normal modes, Varsanyi notation is used.^35^ Abbreviations: oop = out-of-plane, ip = in-plane, def = deformation, disp = displacement, str = stretch, *τ* = torsion

*anti*-3CP		*syn*-3CP		Mode description
*ν̃*_exp_	*ν̃*_DFT_	*ν̃*_exp_	*ν̃*_DFT_	
229	228	228	228	*ν*_10b_
247	245	246	244	C–O, C–Cl bend
315	339	315	338	*τ*_OH_
412	404	412	404	C–Cl str
442	439	444	442	ip O–C–C bend
455	449	—	446	*ν*_16a_
493	490	496	488	Overtone of 247/246
528	530	529	531	*ν*_6a_
540	568	541	567	oop bend
574	584	—	582	oop ring bend, C–C twist
595	—	598	—	*τ*_OH_^2^
672	680	672	683	*ν*_4_
686	687	688	690	*ν*_6b_
763	766	773	777	oop C–H bend
863	—	839	—	*τ*_OH_^3^
881	878	879	884	oop C–H bend
893	887	893	885	Ring def, C–Cl str
1000	1000	1000	1000	*ν*_12_
1072	1075	1070	1073	Ring def, C–Cl disp
1088	1095	1090	1096	C–H bend
1102	—	1104	—	Combination 686 + 412
1158	1167	1152	1165	C–H bend (all)
1170	1176	1178	1175	C–H bend (C4, C5, C6)
1189	—	1192	—	?
1257	1260	1251	1258	*ν*_13_
1293	1308	1299	1322	ip C–H bend, O–H bend
1315	1325	1310	—	ip C–H bend
1474	1471	1442	1454	ip C–H bend, ring def
1484	1489	1488	1500	ip C–H bend
1592	1603	—	1603	C=C ring str
1606	1615	1598	1617	C=C ring str

	6.9		8.6	*Mean absolute deviation*

Up to 800 cm^−1^, the spectrum of both conformers is populated by bands corresponding to both in-plane and out-of-plane bending vibrations. Above 800 cm^−1^, the spectrum is dominated by in-plane modes primarily involving C–H bending, while the highest energy bands correspond to C–C and C–O stretching modes.

In the low-frequency range, we immediately find the largest mismatch between theory and experiment in a very intense predicted band for the OH torsional (*τ*_OH_) mode. Given the good match between other experimental and calculated modes, this predicted band must be assigned to the experimental band observed at 315 cm^−1^ for both conformers. This experimental value is in good agreement with literature values.^12^ The mismatch in both frequency and intensity also mirrors observations for *τ*_OH_ in other jet-cooled phenol derivatives.^24^

Although the only structural difference between the two conformers is the orientation of the hydroxyl group, several bands across the whole spectrum exhibit conformer-specific frequencies. The lowest frequency mode differing significantly between the two conformers is an out-of-plane C–H bending mode observed at 763/773 cm^−1^. An even more dramatic difference of 24 cm^−1^ is observed for the band at 863/839, cm^−1^, a vibration mainly involving out-of-plane bending of the C(2)–H group. Further to the blue, another major difference involves the band pairs at 1257/1251 and 1293/1299 cm^−1^. Experimentally, *anti*-3CP shows a doublet of bands (1257/1293 cm^−1^) with the high-frequency partner the most intense; for *syn*-3CP (1251/1299 cm^−1^) the intensity distribution is reversed. The computations accurately predict these intensity patterns. In both cases, the lower frequency mode has predominantly a C–O stretching character, which matches very well the frequency of the analogous *ν*_13_ mode of phenol.^36,37^ Finally, in the 1400–1500 cm^−1^ range, the *anti* conformer spectrum shows an intense band at 1474 cm^−1^ and a weaker one at 1435 cm^−1^. In contrast, the *syn*-spectrum shows two bands of similar intensities at 1488 and 1442 cm^−1^, respectively. The computations predict two closely spaced bands for *anti*-3CP, (at 1481 and 1464 cm^−1^) that combine into a single band at 1481 cm^−1^, whereas *syn*-3CP displays two bands at 1492 and 1447 cm^−1^, respectively.

While the symmetry reduction by halogenation prevents one from making an ‘elegant’ symmetry-based assignment for the majority of the vibrational modes, several vibrations show close similarity to the well-characterized phenol modes (for an overview of the relevant frequencies please see ESI†).^36,37^ The 229/228 cm^−1^ mode is an analogue of the phenol *ν*_10b_ mode,^35^ an out-of-plane ring deformation. The 455/455 cm^−1^ vibration can be thought of as a phenol *ν*_16a_ mode analogue, also an out-of-plane displacement. The phenol *ν*_6a_ and *ν*_6b_ modes, in-plane ring deformations, are found at 528/529 and 686/688 cm^−1^. The *ν*_4_ analogue, a concerted out-of-plane motion of the hydrogen atoms at positions 2, 4, and 6, is observed at 672 cm^−1^ for both conformers. The bands observed at 1000 cm^−1^ for both conformers can readily be assigned to ‘ring breathing’ modes, at a near-identical frequency as is observed for phenol.^36,37^

Two bands of particular interest for the halogen substitution are the ones observed at 412/412 and 891/893 cm^−1^. DFT predicts modes at 402/402 cm^−1^ and 882/881 cm^−1^ both involving a significant C–Cl stretch contribution, with the higher frequency mode having a significant ring-breathing character. The ring-breathing deformation is not unlike that resulting from coupling of the C–Cl motion with a skeletal vibration, which was shown to give rise to two C–Cl stretching bands in chlorinated benzene derivatives.^35^ This observation is supported by the considerable red-shift of the C–Cl stretch with respect to its usual frequency.^38,39^

Out of the vibrations showing a close resemblance to the phenol modes, the *ν*_16a_ and *ν*_6b_ analogues show significant blue shifts with respect to the phenol bands of 41 and 68/70 cm^−1^, respectively. In contrast, the *ν*_10b_ and *ν*_6a_ analogues exhibit an only negligible blue shift of 2–4 cm^−1^, which is well within the spectral bandwidth of FELIX.^37^ Inspection of the displacement vectors suggests that highly delocalised vibrations show limited influence of chlorine substitution, unless the C–Cl bond is involved explicitly or the motion of the other atoms is perturbed by the Cl presence. For the *ν*_16a_ mode, a marked difference between the phenol mode and the 3CP analogue is the lack of Cl participation in the vibration, which otherwise involves a significant contribution from the C3 substituent (hydrogen) in phenol. In the *ν*_6b_ analogue, a strong C–Cl stretching motion accompanies the ring deformation. Conversely, in phenol, *ν*_10b_ has participation of C3, but in 3CP the Cl is not involved. A similar observation can be made for the *ν*_6a_ mode where significant delocalisation of the vibration prevails over the local effect of chlorine substitution.

The highest frequency bands observed in the experimental spectra around 1600 cm^−1^ can be assigned to C–C ring stretching modes. Although only experimentally resolved for the *anti* conformer, for both species the calculations predict two such modes, differing only by the specific C–C bond involved.

Not all bands, however, can be assigned purely based on the results of the harmonic calculations. We note an intriguing progression of bands at 595/598, 863/839 and 1189/1192 cm^−1^ for *anti*- and *syn*-3CP, which have no DFT counterpart in the harmonic approximation. We speculate that these bands could be the overtones of the OH torsional mode, for which the fundamental was observed at 315 cm^−1^. It is well-known that such modes are strongly anharmonic.^24^ Anharmonic calculations (Fig. S3 of the ESI†) place *τ*_OH_ at 328/311 cm^−1^ and its first overtone at 629/606 cm^−1^. Based on these calculated frequencies of the fundamental and the first overtone, we can extract an anharmonicity parameter and estimate the frequencies of the higher order overtones, yielding the second and third overtone frequencies of 903/885 and 1150/1148 cm^−1^. Using the experimental values in place of the DFT-derived ones gives 840/849 for the second overtone, and 1050/1068 for the third one. However, since these estimated values do not match closely with the experimental results, we decided to perform additional calculations. To simultaneously describe the OH torsional modes for both conformers, a 1-dimensional torsional model was constructed using the moment of inertia calculated by Manocha *et al.*^12^ This torsional model places the OH torsion fundamental and its overtones at 312/315, 600/606, 862/856, and 1111/1191 cm^−1^. While not in perfect agreement with the observed frequencies, these results support our tentative assignment of the second overtone bands. It is important to note, however, that the wavefunctions for the higher vibrational states are highly delocalised (shown in Fig. S5, ESI†) and thus the *syn* and *anti* labels do not perfectly reflect the geometries of the molecules involved. Another possibility for the assignment of those modes would be combination bands. For the 1189/1192 band, the only contenders based on a frequency match are combination mode calculated at 1189/1193 cm^−1^ (Fig. S3, ESI†). While the intensity of the calculated 1193 cm^−1^ is appreciable, the intensity of the 1189 cm^−1^ band is near-negligible. As such, we choose to refrain from assigning that peak.

### 3.2 Spectroscopy of 3-fluorophenol

**Fig. 4:**
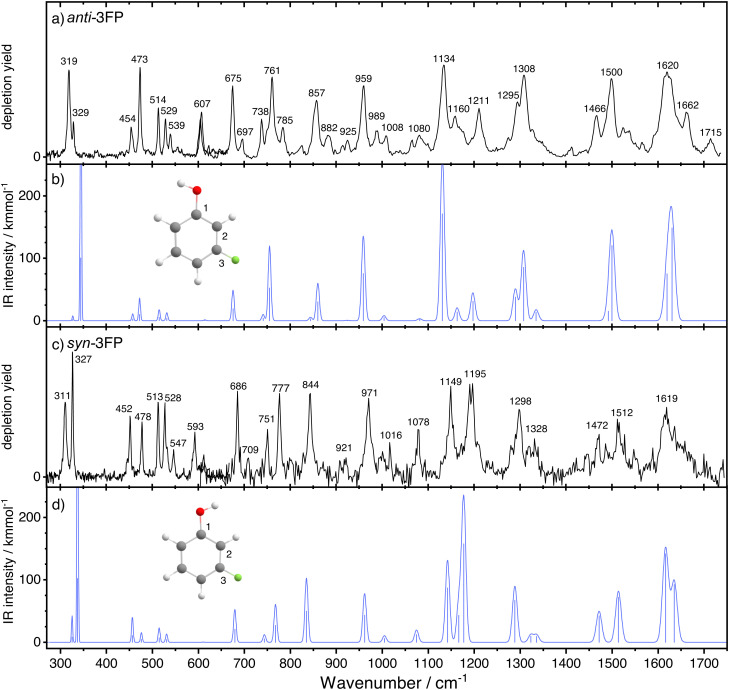
The experimental (a) and (c) and simulated (b) and (d) spectra of *anti*- and *syn*-3FP, respectively. In panels (b) and (d), the blue line represents the scaled harmonic spectrum. The spectrum of *syn*-3FP was multiplied by a factor 2 in the region above 600 cm^−1^ to facilitate comparison between the two conformers.

Fig. 4 shows the conformer-specific infrared spectra recorded for *anti*- (panel a) and *syn*-3FP (panel c). Each experimental spectrum is compared to a DFT-calculated spectrum (panels b and d).

Overall, the harmonic calculated spectra are again in good agreement with the experiments, with the intensities and band positions being accurately reproduced. Similar to 3CP, an exception is formed by the OH torsional mode, for which the frequency is overestimated by the harmonic calculations. A detailed assignment of the observed vibrational bands is presented in Table 2, while a general discussion is presented below. The *anti*/*syn* convention used while discussing the band line positions for 3CP is used here as well.

**Table 2:** Observed (*ν̃*_exp_) and calculated (*ν̃*_DFT_) band frequencies in cm^−1^ in the IR-UV ion dip spectra of 3FP and their assignments. For vibrations which are analogous to phenol normal modes, Varsanyi notation is used.^35^ Abbreviations: oop = out-of-plane, ip = in-plane, def = deformation, str = stretch, *τ* = torsion, exp = experimental

*anti*-3FP		*syn*-3FP		Mode description
*ν̃*_exp_	*ν̃*_DFT_	*ν̃*_exp_	*ν̃*_DFT_	
319	345	311	337	*τ*_OH_
329	325	327	325	C–O, C–F bend
454	456	452	457	*ν*_16a_
473	471	478	477	C–O, C–F bend
514	513	513	514	*ν*_6b_
529	529	528	531	*ν*_6a_
539	—	547	—	*τ*_OH_ (exp) + 223/223(DFT)
607	—	593	—	*τ*_OH_^2^
675	673	686	680	*ν*_4_
738	742	751	744	Ring def, C–F str
761	756	777	768	oop C–H bend
785	—	—	—	*τ*_OH_ + 473
857	861	844	836	*ν*_17b_
925	924	—	—	oop C–H bend
959	959	971	959	Ring def, C–O str
989	—	—	—	*τ*_OH_^3^
1008	1006	1016	1006	*ν*_12_
1080	1082	1078	1076	ip C–H bend (C4 and C6)
1134	1131	1149	1142	ip C–H bend, C–O, C–F str
1160	1164	—	1166	ip C–H, O–H bend
1211	1198	1195	1177	ip C–H, O–H bend, C–F str
1295	1288	1298	1288	*ν*_13_
1308	1307	1328	1331	ip C–H bend, C–O str
1466	1492	1472	1472	H–O–C bend
1500	1500	1512	1513	Ring def, O–H bend
1620	1628	1619	1618	C–C stretch
1662	—	—	—	Combination *ν*_16a_ + 1211
1715	—	—	—	*ν*_17b_^2^

	5.3		6.7	*Mean absolute deviation*

Within the investigated spectral range, the lowest frequency bands are the doublet features observed at 319 and 329 cm^−1^ for anti-3FP and at 311 and 327 cm^−1^ for *syn*-3FP, respectively. The calculations yield an in-plane C–O, C–F bending mode at 325 cm^−1^ for both conformers, matching the higher-frequency components of the observed features. This suggests that the lower-frequency components correspond to the OH torsional mode, in line with the assignments by Manocha *et al.*,^12^ but crucially reversing them. Manocha *et al.* made no use of an conformer-specific detection scheme and observed the bands at 311 and 319 cm^−1^ simultaneously. Their stability-based argument to assign the 319 cm^−1^ band to the *anti* conformer is here disproven with the IR-UV ion-dip experiments. The assignment of the UV spectra, originally based on dispersed fluorescence,^13,14^ is confirmed by the good agreement of the vast majority of frequencies in our IR spectra with the results of the DFT calculations. Consequently, we assign the 319 cm^−1^ band to *τ*_OH_ of the *anti* conformer and the 311 cm^−1^ band to its *syn* counterpart. The assignment is further strengthened by the positions of the assigned overtones at 607/593 cm^−1^, and 989/921 cm^−1^.

A total of twelve vibrations show a frequency difference of 8 cm^−1^ or more between the two conformers, against only five such vibrations identified for 3CP. A significant number of these, particularly in the range above 1000 cm^−1^, contain contributions from C–F stretching, suggesting that fluorination considerably affects the vibrational levels. Out of the twelve, four modes are observed at a higher frequency for the *anti* conformer than for its *syn* counterpart. Apart from the aforementioned OH torsion fundamental, these include the 857/844 cm^−1^ bands corresponding to an out-of-plane C–H bending mode analogous to *ν*_17b_ of phenol. While for the *anti* conformer the calculated displacement vectors show an out-of-plane motion of the hydrogen atoms at positions 2, 4, and 6, for the *syn* conformer the hydrogen atom at position 2 – between the OH and F substituents – is stationary. The orientation of the hydroxyl group therefore appears to have an influence on both frequency and normal coordinate of the vibration. The final mode of the group is the 1211/1195 cm^−1^ band corresponding to an in-plane C–H and O–H bending, with some C–F stretching character. Importantly, while we do observe a large disparity between the frequencies of other modes involving C–F stretching, it appears that this mode is not strongly affected.

Yet again, we can make a direct comparison between a number of the 3-fluorophenol modes and their phenol counterparts. The *ν*_16a_ mode (454/452 cm^−1^) is blue-shifted by about 50 cm^−1^ upon halogenation, a shift identical with that observed for 3-chlorophenol. *ν*_4_, observed at 675/686 cm^−1^, shows a minor red shift for the both conformers of 3CP and for *anti*-3FP and no shift whatsoever for *syn*-3FP. This is to be expected as in phenol there is no involvement of C3 or H3 and, consequently, the frequency of *ν*_4_ ought not to be significantly affected by *meta*-substitution. The previously discussed *ν*_17b_ analogue is significantly red-shifted with respect to the phenol mode (by 24/37 cm^−1^). Unlike for 3CP, where there is virtually no shift, the *ν*_12_ mode is slightly blue shifted with respect to phenol. Finally, the analogue of the *ν*_13_ mode is significantly blue shifted for 3FP by over 30 cm^−1^ for both conformers.

Not all features in the spectrum can be straightforwardly assigned. Due to the increase in spectral bandwidth with increasing frequency, similarly to 3-chlorophenol the C–C stretching modes cannot be resolved. Apart from these vibrations, in the spectrum of the *anti* conformer we observe several modes which are not accounted for by harmonic calculations. Based on the experimental frequencies, we tentatively assign the 1715 cm^−1^ mode as the first overtone of *ν*_17b_. An alternative assignment, brought forward by anharmonic calculations, is that to the overtone of a weak band predicted at 848 cm^−1^ (scaled harmonic frequency) which is not resolved in our experimental spectrum. The feature observed at 785 cm^−1^ in the *anti* conformer spectrum is most likely a combination mode. Based on the closest frequency match, we tentatively assign it as a combination of OH torsion and the 473 cm^−1^ bending mode. The 1662 cm^−1^ band was assigned following analogous reasoning.

### 3.3 Discussion

What becomes immediately apparent by directly comparing the spectra of the two molecules is that their infrared activities are very similar, with a large number of bands populating the spectral range of interest (see Fig. 2). As mentioned in Section 3.2, 3FP shows a larger number of conformer-specific frequencies than its chlorinated counterpart.

Both 3CP and 3FP exhibit a number of modes which can be compared with the vibrations of phenol. In the case of 3CP, there appears to be a clear correlation between heavy atom substitution and a considerable shift in the frequency of vibrations directly affected by substitution. For 3FP, this relationship is less clear, however overall we see significant deviation between the 3FP and phenol modes. One indirect effect of *meta*-halogenation on the infrared spectrum is the shift in frequencies of the *ν*_13_ mode analogues, which is the (primarily) C–O stretching mode in phenol. While for 3CP we observe a minor red shift, the *ν*_13_ vibration of 3FP is blue-shifted with respect to phenol by over 30 cm^−1^ for both conformers. This discrepancy can be explained by the strong inductive electron-withdrawing effect of fluorine. Fluorine is a strong *σ* electron withdrawing substituent and thus, with a minor decrease in the C–O bond length caused by fluorination,^15^ the stretching frequency increases.

For any experiment aimed at studying interconversion dynamics, accurate knowledge of the potential energy surface describing the torsional modes is of high interest. The separation of the torsional vibrational modes of 3FP observed by Manocha *et al.*^12^ is confirmed in the current experiments, like the lack thereof for 3CP, but the assignment we make is the reverse. Manocha *et al.* suggested that the separation in the OH torsional modes of 3FP is due to a difference in the potential energy minima of the two conformers. In their computations, they found that *syn*-3FP is more stable, but more recent calculations at various levels of theory consistently find that the *anti* conformer is the more stable one.^15,18^ In line with these results, the current calculations place the *anti*-3FP conformer energy below its *syn* counterpart, with an energy difference of 55 cm^−1^, lower than the 70 cm^−1^ found by Moreira *et al.*^18^ Simultaneously, our calculated energy difference of 2 cm^−1^, (well below the expected accuracy of the current level of theory) between the two conformers of 3CP is consistent with the lack of separation between the two torsional modes.

**Fig. 5:**
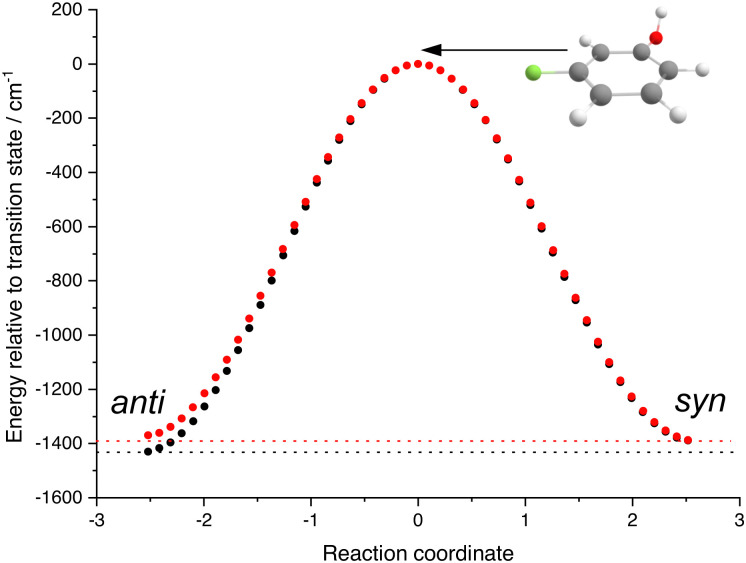
Internal reaction coordinate (IRC) calculation result for 3FP (black dots) and 3CP (red dots). The horizontal dotted lines correspond to the energies of the more stable conformer for each species. The geometry of the transition state is shown above the curve.

Given the accurate predictions of the current computations for the experimental IR spectra, we calculated the potential energy surfaces of the torsional modes of 3FP and 3CP (Fig. 5). The results are presented referenced to the interconversion barrier, which is found to lie at ∼1400 cm^−1^. This value implies that a direct, ladder-like IR excitation of *τ*_OH_ is an improbable route for successful interconversion, given the need to (a) absorb five quanta and (b) the significant anharmonicity of the potential, illustrated by the bands assigned to overtones of *τ*_OH_. However, the observation of interconversion in matrices suggest that an alternative pathway is available, likely including excitation of a higher-frequency mode, followed by intramolecular vibrational distribution. This pathway could be studied by several approaches. First, isomer population changes and barrier heights can be experimentally studied *via* hole-filling spectroscopy.^40,41^ These studies rely on laser-induced isomerization, followed by collisional cooling of the formed isomers, yielding sharp transition lines. However, in a dynamics experiment, such collisions, which typically take place at much larger timescales, are likely irrelevant. Therefore, it will be of interest to also probe the UV spectra directly after IR excitation, monitoring the (transiently) populated vibrational states. Probing dynamics of such processes on their natural timescale requires short laser pulses, which are characterised by a much larger bandwidth than the nanosecond pulses employed in this experiment. The increased bandwidth reduces spectral resolution, thus rendering conformer separation based on resonant ionisation impossible. Therefore, we are currently working on the introduction of electrostatic selection techniques for these experiments, that yield conformer-selected molecular beams.^42^ Secondly, the use of alternative detection techniques, such as velocity-map imaging would enable obtaining spectral information (in the form of a photoelectron spectrum) with just one excitation wavelength. Finally, a crucial requirement for time-resolved experiments is the ability to isolate single IR picosecond pulses rather than a 10 μs FEL pulse train. This was recently demonstrated, resulting in micropulse energies exceeding 100 μJ,^43^ which should be sufficient to saturate the majority of vibrational transitions. Experiments aimed at exploiting the combination of these technical improvements are currently in preparation in our Laboratory.

## 4 Conclusions

In this work we presented IR ion-dip spectra of gas-phase, jet-cooled 3CP and 3FP encompassing the majority of the fingerprint region. With the aid of DFT calculations and thanks to conformer-selectivity of the method we were able to assign the large majority of bands. Our assignment revises a previous one of the OH torsional modes of 3FP, attributing the higher frequency vibration to the *anti* conformer.

Our simulated spectra, calculated at the B3LYP/def2-tzvp level of theory, provide a good match for the experimental results within the harmonic approximation, with the exception of the OH torsional mode. Alternative approaches, like Born–Oppenheimer Molecular Dynamics (BOMD) calculations were suggested for phenol derivatives and could be implemented should a higher degree of accuracy be required to identify hydroxyl group torsional modes.^10,24^ Furthermore, simple anharmonic correction seems to be of limited use in assigning modes falling beyond the scope of the harmonic approximation, apart from the relatively well reproduced first overtones of the OH torsional mode. A simple, one-dimensional harmonic oscillator model of the OH torsional mode gives strength to the identification of several higher overtones of the torsional mode.

## Data availability

The data supporting this article have been included as part of the ESI.†

## Conflicts of interest

There are no conflicts to declare.

## Supplementary Material

CP-027-D4CP04352A-s001

CP-027-D4CP04352A-s002
