# Correction: Gas-phase, conformer-specific infrared spectra of 3-chlorophenol and 3-fluorophenol

**DOI:** 10.1039/d5cp90068a

**Published:** 2025-04-02

**Authors:** Olga A. Duda, Alexander K. Lemmens, Gerrit C. Groenenboom, Daniel A. Horke, Joost M. Bakker

**Affiliations:** a Institute for Molecules and Materials, HFML-FELIX Laboratory, Radboud University Toernooiveld 7 6525 ED Nijmegen The Netherlands joost.bakker@ru.nl; b Institute for Molecules and Materials, Radboud University Heijendaalseweg 135 6525 AJ Nijmegen The Netherlands; c Chemical Sciences Division, Lawrence Berkeley National Laboratory Berkeley California 94720 USA

## Abstract

Correction for ‘Gas-phase, conformer-specific infrared spectra of 3-chlorophenol and 3-fluorophenol’ by Olga A. Duda *et al.*, *Phys. Chem. Chem. Phys.*, 2025, https://doi.org/10.1039/d4cp04352a.

The authors regret the omission of one of the authors, Alexander K. Lemmens, from the original article. The corrected list of authors and affiliations for this paper is as shown here.

The Royal Society of Chemistry apologises for these errors and any consequent inconvenience to authors and readers.

