# Supplementary material for: Gas-phase, conformer-specific infrared spectra of 3-chlorophenol and 3-fluorophenol
Source: Phys Chem Chem Phys. 2025 Feb 20;27(15):7565–73. doi: 10.1039/d4cp04352a (PMC11869563; doi:10.1039/d4cp04352a)
Supplement: CP-027-D4CP04352A-s001 [file CP-027-D4CP04352A-s001.pdf]

Supplementary Information (SI) for Physical Chemistry Chemical Physics.
This journal is © the Owner Societies 2025

```
Frequency calculation:
#p B3LYP/Def2TZVP opt=(CalcAll,MaxCycle=100,MaxStep=100,verytight)
Freq=Anharmonic pop=(full,NBO) IOp(6/7=3)

anti-3CP xyz coordinates:
0, 1
C              -0.5596300000      1.4097770000      0.0000000000
C              -0.8358610000      0.0473940000      0.0000000000
C               0.7692490000      1.8130050000      0.0000000000
H               1.0049340000      2.8695490000      0.0000000000
C               0.1716790000     -0.9036600000      0.0000000000
C               1.8008080000      0.8823570000      0.0000000000
H              -0.0532390000     -1.9603600000      0.0000000000
H               2.8343700000      1.2095410000      0.0000000000
C               1.4979320000     -0.4774450000      0.0000000000
H              -1.3645220000      2.1300770000      0.0000000000
O               2.4580200000     -1.4457570000      0.0000000000
Cl             -2.4989730000     -0.4866160000      0.0000000000
H               3.3317880000     -1.0388510000      0.0000000000

syn 3CP xyz coordinates:
0, 1
C               0.5591320000      1.4150320000      0.0000000000
C               0.8320250000      0.0554040000      0.0000000000
C              -0.7722350000      1.8211420000      0.0000000000
H              -1.0029220000      2.8788380000      0.0000000000
C              -0.1831760000     -0.8922000000      0.0000000000
C              -1.8069110000      0.8984250000      0.0000000000
H               0.0590130000     -1.9476060000      0.0000000000
H              -2.8423120000      1.2103770000      0.0000000000
C              -1.5081950000     -0.4624750000      0.0000000000
H               1.3653670000      2.1338850000      0.0000000000
O              -2.5542010000     -1.3370820000      0.0000000000
H              -2.2262360000     -2.2434920000     -0.0000010000
Cl              2.4915790000     -0.4910190000      0.0000000000

anti-3FP xyz coordinates:
0, 1
C              -1.2446410000      1.0700390000      0.0000000000
C              -1.1883030000     -0.3140530000      0.0000000000
C              -0.0412870000      1.7644220000      0.0000000000
H              -0.0501530000      2.8467280000      0.0000000000
C              -0.0014260000     -1.0204920000      0.0000000000
C               1.1751350000      1.0922320000      0.0000000000
H               0.0030620000     -2.1011360000      0.0000000000
H               2.1067860000      1.6462910000      0.0000000000
C               1.1917130000     -0.3018340000      0.0000000000
H              -2.2003380000      1.5742280000      0.0000000000
O               2.3486510000     -1.0232180000      0.0000000000
F              -2.3444800000     -1.0107730000      0.0000000000
H               3.1046090000     -0.4252870000      0.0000020000

syn-3FP xyz coordinates:
0, 1
C               1.2597650000      1.0567310000      0.0000000000
C               1.1806070000     -0.3228580000      0.0000000000
C               0.0647860000      1.7716450000      0.0000000000
H               0.0943760000      2.8536340000      0.0000000000
C              -0.0229730000     -1.0069190000      0.0000000000
C              -1.1625460000      1.1256820000      0.0000000000
H              -0.0255190000     -2.0899380000      0.0000000000
H              -2.0913650000      1.6791300000      0.0000000000
C              -1.2039780000     -0.2676640000      0.0000000000
H               2.2235540000      1.5453220000      0.0000000000
O              -2.4313510000     -0.8613170000      0.0000000000
H              -2.3339130000     -1.8201650000      0.0000010000
F               2.3210790000     -1.0463510000      0.0000000000
```
