# Supplementary material for: Gas-phase, conformer-specific infrared spectra of 3-chlorophenol and 3-fluorophenol
Source: Phys Chem Chem Phys. 2025 Feb 20;27(15):7565–73. doi: 10.1039/d4cp04352a (PMC11869563; doi:10.1039/d4cp04352a)
Supplement: CP-027-D4CP04352A-s002 [file CP-027-D4CP04352A-s002.pdf]

Supplementary Information (SI) for Physical Chemistry Chemical Physics.
This journal is © the Owner Societies 2025

Gas phase conformer-specific far-infrared spectra of 3-chlorophenol and 3-fluorophenol

Olga. A. Duda,[1] Gerrit C. Groenenboom,[2] Daniel A. Horke,[2] and Joost M. Bakker[1]

[1] *Radboud University, Institute for Molecules and Materials, HFML-FELIX, Toernooiveld 7, 6525 ED Nijmegen*
[2] *Radboud University, Institute for Molecules and Materials, Heijendaalseweg 135, 6525 AJ Nijmegen, The Netherlands*

Supporting Information

I. Resonance-Enhanced MultiPhoton Ionisation (REMPI) spectra

The REMPI spectra of 3-chlorophenol and 3-fluorophenol are shown below.

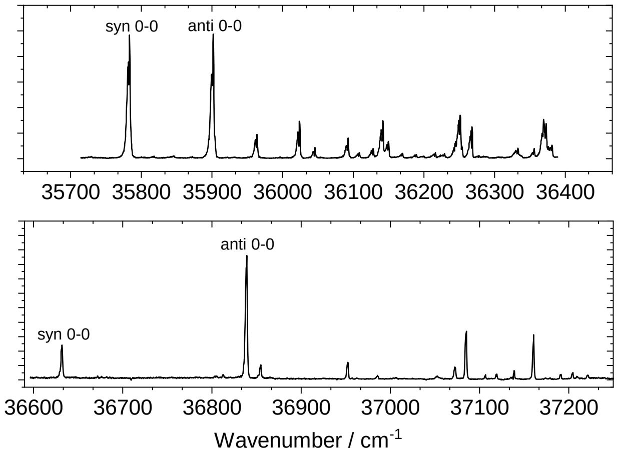

FIG. S1. REMPI spectra of 3-chlorophenol (top) and 3-fluorophenol (bottom). The origin bands, used in the IR-UV ion dip scheme, are labelled.

II. Raw infrared spectra

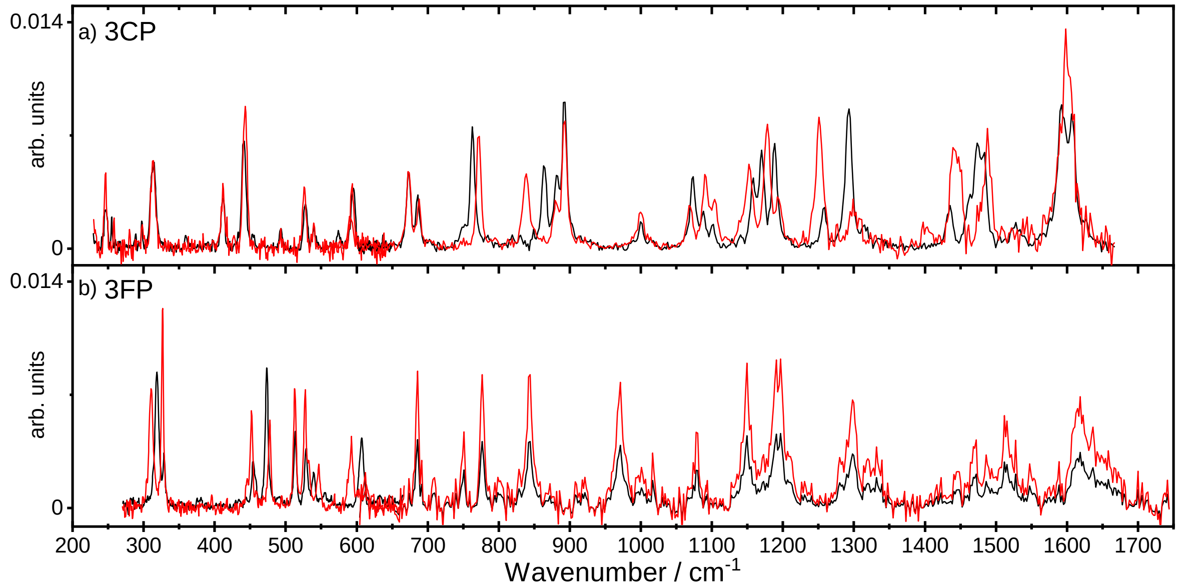

FIG. S2. Isomer-resolved infrared spectra of 3CP and 3FP presented with power correction, without vertical scaling.

III. Anharmonic calculations

The anharmonic calculations were obtained by using the freq=anharmonic keyword in Gaussian16.[?] The anharmonic correction utilizes the Rayleigh-Schrödinger perturbation theory in order to calculate the anharmonic infrared frequencies.[?] The calculated frequencies were convolved with a Gaussian curve with FWHM=0.5% of the central wavelength. The convolved spectra are shown in Figures **??** and **??**.

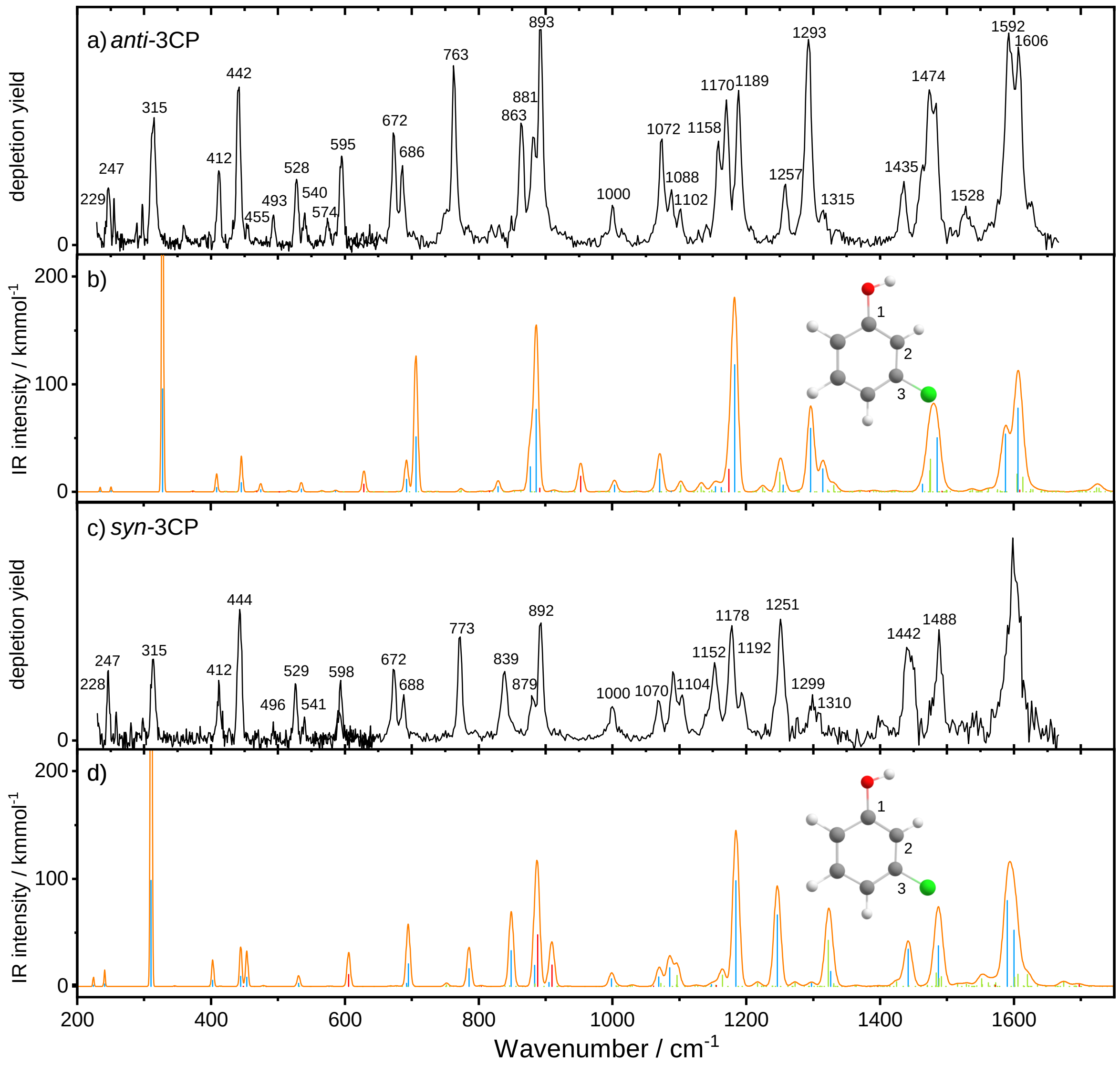

FIG. S3. The infrared spectrum of the two isomers of 3-chlorophenol compared to the simulated spectra. The orange trace in panels b) and d) corresponds to the total convolution of all of the calculated modes. The stick spectra representing individual vibrational modes are colour-coded as follows: fundamental - blue, overtones - red, combination - green.

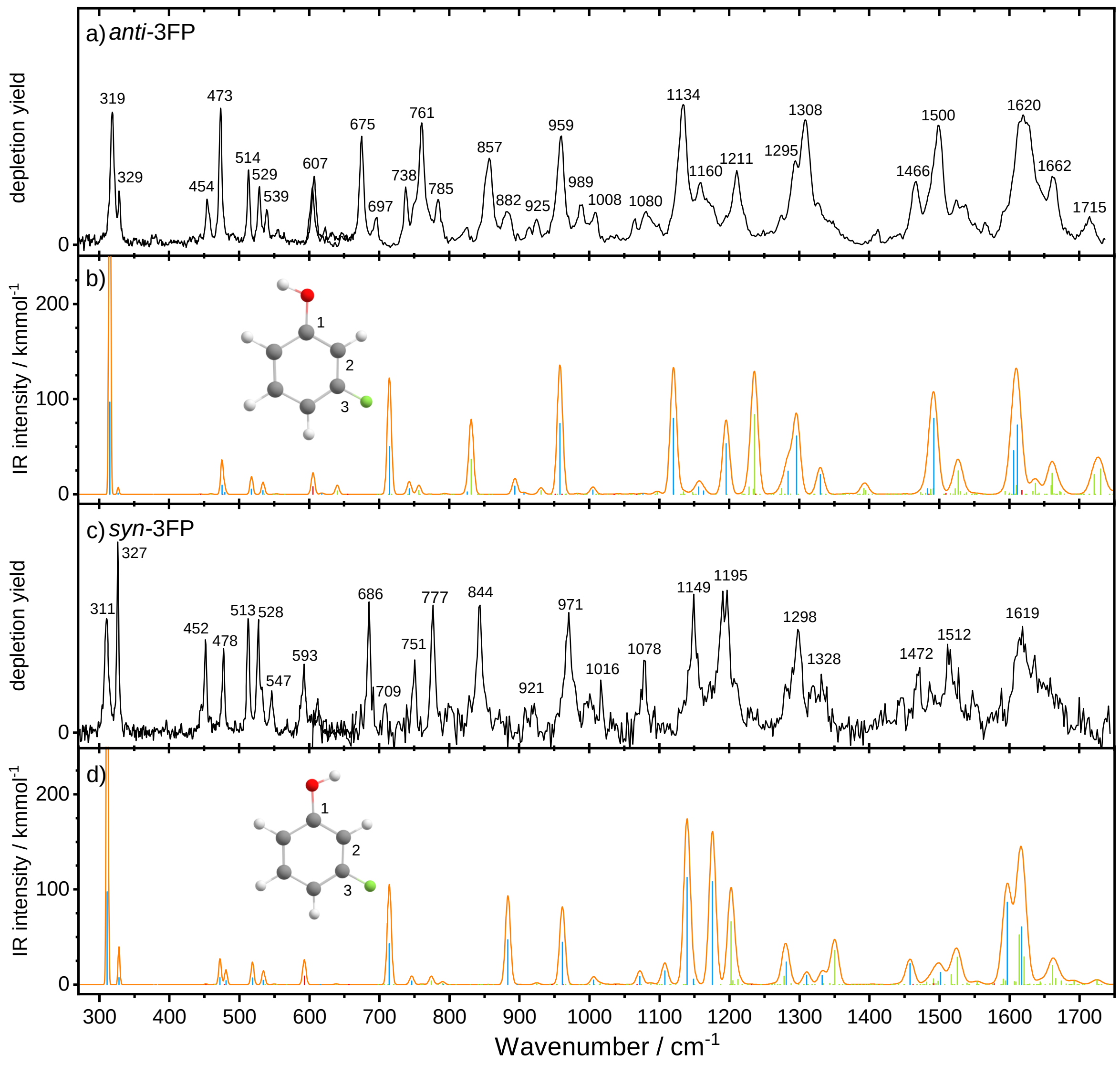

FIG. S4. The infrared spectrum of the two isomers of 3-fluorophenol compared to the simulated spectra. The orange trace in panels b) and d) corresponds to the result of the anharmonic calculations. The stick spectra representing individual vibrational modes are colour-coded as follows: fundamental - blue, overtones - red, combination - green.

IV. One dimensional torsional model for 3CP

The torsional wave functions for 3CP were computed for the potential shown in Figure 5. We took the one-half effective inverse moment of inertia from table 1 of Manocha *et al.* [12]. We only included the constant $F_0 = 22.4869$ cm^{-1} term and neglected the torsion-angle dependent terms, which are more than a factor thousand smaller. The potential was scaled by a factor 0.969 in order to reproduce the observed torsional frequency of 315 cm^{-1} for the *syn* conformer. It was expanded as $V(\phi) = \sum_{n=0}^{6} \cos(n\phi)c_n$, with $c_0, \ldots, c_6 = 659.31, 5.70, -670.60, 3.14, 13.63, -0.66, 0.97$ cm^{-1}. We use a rotational basis of $\{\cos n\phi$, with $n = 0, 1, \ldots, 30\}$ for the wave functions shown in blue in Figure **??** (columns 1 and 3) and a rotational basis of $\{\sin n\phi$, with $n = 1, 2, \ldots, 30\}$ for the functions in red (columns 2 and 4). In these calculations, the lowest *syn* energy level is 15 cm^{-1} lower in energy than the lowest *anti* energy level.

It can be seen that with the increase in the vibrational quantum number, the wavefunctions become more delocalised.

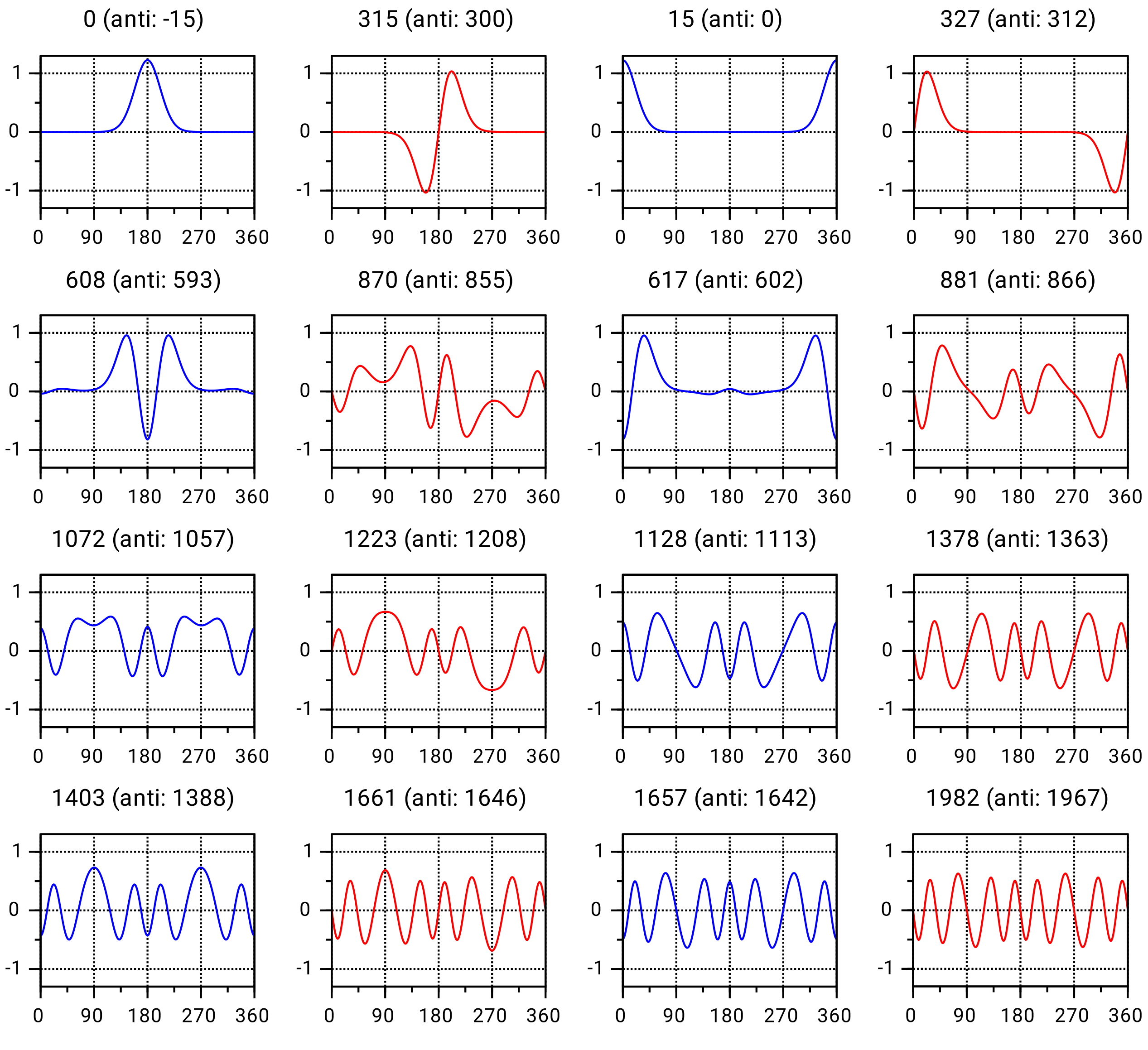

FIG. S5. Wavefunctions for 3CP calculated on the basis of the 1D torsional potential. The two left columns show the *syn* isomer states, while the two right columns show the *anti* isomer states. The *syn* ground state vibrational wave function, located at torsion angle $\phi = 180°$ is shown in the upper left panel. The *anti* ground state vibrational wave function, located at $\phi = 0°$ is shown in the third panel of the top row. The energies (in cm^{-1}) relative to the *syn* ground state are given in the titles of the panels in cm^{-1}. In parentheses the energies relative to the *anti* ground level level are shown.

V. Natural Bond Order calculations - charge distribution

The charge distribution as calculated using the pop=NBO keyword as implemented in Gaussian16 is given below.

Atom	No	Charge	Core	Valence	Rydberg	Total
C	1	-0.26079	1.99900	4.24019	0.02160	6.26079
C	2	-0.01277	1.99861	3.98722	0.02694	6.01277
C	3	-0.16931	1.99921	4.15053	0.01957	6.16931
H	4	0.21366	0.00000	0.78515	0.00120	0.78634
C	5	-0.28082	1.99886	4.25809	0.02387	6.28082
C	6	-0.29621	1.99904	4.27719	0.01998	6.29621
H	7	0.23231	0.00000	0.76576	0.00193	0.76769
H	8	0.20615	0.00000	0.79238	0.00147	0.79385
C	9	0.30331	1.99874	3.67744	0.02051	5.69669
H	10	0.22466	0.00000	0.77392	0.00142	0.77534
O	11	-0.63871	1.99978	6.61602	0.02291	8.63871
Cl	12	0.00537	9.99963	6.97877	0.01623	16.99463
H	13	0.47316	0.00000	0.52476	0.00207	0.52684
Total	=	0.00000	23.99288	41.82742	0.17970	66.00000

VI. Phenol vibrations

The table below contains the frequencies of the phenol vibrations relevant for the discussion of the assigned 3CP and 3FP modes. All the modes are labelled using the Varsanyi notation and their frequencies are taken from *Roth et al.*[? ?]

Mode	Frequency [cm^{-1}]
10b	225
τ(OH)	310
16a	404
6a	526
6b	618
4	686
17b	881
12	999
13	1261

VII. Overtone frequency estimations

The tables below summarise the frequencies (in cm^{-1}) calculated for the second and third overtones of the OH torsional mode for the conformers of 3CP and 3FP based on the harmonic frequency ω and anharmonicity parameters x, derived using the fundamental and the first overtone via $\tilde{\nu}_{0\leftarrow 1} = \omega(1-2x)$ and $\tilde{\nu}_{0\leftarrow 2} = \omega(2-6x)$. Abbreviations used are TM = torsional model; est = Taylor expansion estimate;

anti-3CP:

Transition	Observed	Estimates based on:		Torsional model
		Experiment ω=350, ωx=17.5	DFT ω=355, ω=13.5	
$0 \to 1$	315	315	328	312
$0 \to 2$	595	595	629	600
$0 \to 3$	863	840	903	862
$0 \to 4$	-	1050	1150	1111

syn-3CP:

Transition	Observed	Estimates based on:		Torsional model
		Experiment ω=347, ωx=16	DFT ω=327, ω=8	
$0 \to 1$	315	315	311	315
$0 \to 2$	598	598	606	606
$0 \to 3$	839	849	885	856
$0 \to 4$	-	1068	1148	1191

anti-3FP:

Transition	Observed	Estimates based on:	
		Experiment ω=350, ωx=15.5	DFT ω=340, ω=12.5
$0 \to 1$	319	319	315
$0 \to 2$	607	607	605
$0 \to 3$	989	864	870
$0 \to 4$	-	1090	1110

syn-3FP:

Transition	Observed	Estimates based on:	
		Experiment ω=340, ωx=12.5	DFT ω=340, ω=14.5
$0 \to 1$	311	311	315
$0 \to 2$	593	593	605
$0 \to 3$	-	846	870
$0 \to 4$	-	1070	1110
